# Development and Application Evaluation of a Nursing Simulation Teaching Information System Based on Hospital Information Systems

**DOI:** 10.1155/2023/6334967

**Published:** 2023-01-14

**Authors:** Lei Zhang, Jing Wu, Jie Yang, Shao-Shi Chen, Jing-Ping Liu, Ping Zhang, Jing Chu, Chen-Ling Luo

**Affiliations:** ^1^School of Nursing, Naval Medical University, Shanghai 200433, China; ^2^The University of Hong Kong-Shenzhen Hospital, Shenzhen, Guangdong 518000, China; ^3^Department of Nursing, The Seventh Affiliated Hospital of Southern Medical University, Foshan, Guangdong 528244, China; ^4^School of Nursing, Southern Medical University, Guangzhou, Guangdong 510515, China

## Abstract

**Background:**

The extensive application of hospital information systems in the current information-driven era suggests that nursing education should focus on information education.

**Methods:**

The newly developed hospital information system was used and evaluated by 544 students to explore the feasibility and necessity of such applications for teaching.

**Results:**

Overall, 97.1% of the students expressed satisfaction, and 96.0% supported simulated information education for nursing. The usability was good, with the system receiving a usability score of 72.625 ± 13.0907. The junior students had a higher score than the sophomores regarding system availability, and the difference was statistically significant.

**Conclusions:**

Students generally had a high degree of satisfaction with the simulated information nursing education system and highly approved of the teaching method. However, the system needs to be upgraded.

## 1. Introduction

In the current technology-driven era, those in the public health field should focus on informatics and information technology tools [1, 2]. The extensive use of hospital information systems (HISs) reflects the influence of information technology on medical and health services. Such systems comprise social technology subsystems for hospitals and involve networks, computers, and other modern technologies to transform various medical data into information [3]. Many studies confirm that HISs improve clinical management at the diagnostic and therapeutic levels and reduce the costs associated with paper medical records and error rates [4, 5].

The largest group of HISs users is nurses, and effective training has a significant positive effect on their perceptions regarding the ease and usefulness of HISs [6]. Moreover, training is usually the most effective among students [7]. The basic requirements that nursing students face are conducting clinical work and improving their practical skills [8], meaning nursing colleges need to be aware of the importance of simulating clinical scenarios in discipline education [9]. Research shows that, compared with the traditional teaching model, simulation teaching improves students' knowledge, confidence, and satisfaction [10]. However, nursing simulation information education based on HISs is relatively rare. The reasons for this may include the complexity of HIS, which involves different business modules from the hospital, or ethical factors, such as patient privacy, which make it difficult to directly apply HISs and data to school teaching.

The International Council of Nurses, the Honorary Society of Nursing International, and many national nursing associations advocate for the integration of information technology into nursing education [10]. Furthermore, the University of Athens has designed and implemented an e-learning system, including learning and practice modules, to provide and consolidate HIS-related knowledge for medical students and proposes that an application of a HIS demonstration program should be attempted in the future to simplify the learning process [11]. The Wright State University Miami Valley School of Nursing was one of the first schools in the United States to provide connectivity via computers in the school's student computer lab to a real hospital information system, achieving positive results [12]. When we search for keywords in the database, such as HISs or nursing information education, we seldom consult the literature of relevant research or search the literature for information on the direct application of HISs to nursing colleges.

In China, nursing simulation information education is still in the exploratory stage [13], and due to the limitations of the main teaching points, it is not practical for most nursing students to access actual HISs during school [14]. To address this issue, our team has cooperated with a digital technology company to develop a nursing simulation teaching information system based on HISs in 2018 and has carried out applied research at Southern Medical University in Guangzhou, China.

## 2. Materials and Methods

### 2.1. Study Design and Setting

**Figure 1:**
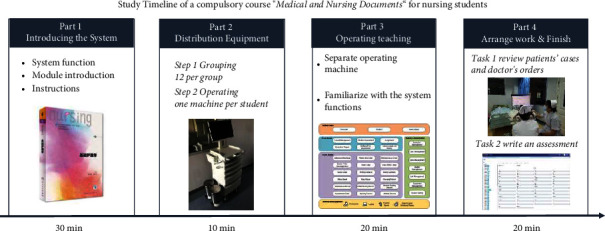
The patient case writing team consists of 12 head nurses from different departments of the university's affiliated hospital and 12 teachers from the school of nursing. Cases are collected in the hospital, and patients' private information is deleted and imported into the system. By the end of 2020, 10 cases would have been imported.

This study was cross-sectional and used a descriptive research method. The development test for the course was arranged as part of the “Medical and Nursing Instruments” section of the required course for nursing students (Figure 1).

### 2.2. Development of the Nursing Simulation Teaching Information System

Developing a simulated information system suitable for nursing experimental teaching is a necessary hardware condition for carrying out nursing information education. The system was self-developed with a digital technology company and supported by the Innovation and Entrepreneurship Education Reform Research Project. It is based on the functional modules and methods of the nurse workstation in clinical HISs and combined with the training objectives and teaching needs of nursing undergraduates. The system is required to do the following: (1) contain all the functions involved in the nurses' work in the HISs; (2) achieve the teaching goal of nursing students to master the common functions of HISs through laboratory simulation training; (3) provide teaching support for internal and surgical nursing, health assessment, basic nursing, and other courses because the system simulation cases cover common diseases in various departments; (4) ensure the simulated cases cover the whole patient process, from admission to discharge, and related information, such as medical records, nursing records, surgical records, doctor's orders, nursing evaluations, temperature sheets, and auxiliary examinations; (5) ensure nursing students are automatically assigned patients and can carry out the simulated management of patients, including medical advice processing, vital sign input, nursing records, nursing evaluation form filling, and execution sheet printing; (6) ensure the clinical case time changes dynamically each day in real time to enhance the sense of imitation and clinical substitution; (7) increase the teaching auxiliary function module, where teachers can use the system to arrange preclass preview tasks, set case time nodes, assign classroom tasks, and the above operations, such as medical advice processing and vital sign input, while students can use the system to conduct self-assessment and mutual evaluation, upload operation video files, and upload postclass work documents; (8) use the enterprise-level cloud system framework.

The system for all business-related processes and operating interfaces is consistent with mainstream HISs. The enterprise cloud system framework is used to ensure the stability, security, compatibility, and scalability of the system when designing the system's technical architecture. The system is compatible with mainstream devices, such as PCs, MACs, iPads, and Android pads, and corresponding apps are being developed to enable teachers and students to log in and use the system at any time. See Figure 1 for system architecture functions.

### 2.3. Participants

From March to May 2021, the second- and third-year nursing students at the university were given the lecture and questionnaire survey, resulting in a total of 544 participants.

### 2.4. Measurement Tool

1. *Nursing Information System Questionnaire*: The questionnaire was modified from the “assessment of the quality of a web-based learning system for nurses” tool [15], with questions added about the students' understanding of the nursing simulation teaching information system and an evaluation of its effects. All the entries were repeatedly revised and completed by three nursing experts. The revised questionnaire was reviewed by two clinical nursing management experts and one associate professor to confirm the validity of its content. The questionnaire had a content validity index of 0.939, and a pilot trial was undertaken with 54 third-year nursing students from March to May 2019. The questionnaire consisted of 20 items, including two open questions regarding the system's benefits and suggestions. The response options correspond to a five-point Likert scale, with “5” representing strongly agree, “4” representing agree, “3” representing neutral, “2” representing disagree, and “1” representing strongly disagree. The Cronbach's *α* coefficient of the questionnaire was determined to be 0.868, indicating good reliability and validity.
2. *System Usability Scale (SUS)*: As a simple and effective measurement tool, the SUS is the most widely used tool in the world for the overall evaluation of feasibility [16–18]. The SUS was originally compiled by John Brooke [19] in 1986 and consists of 10 items, including five positive description items and five negative description items. In 2008, Bangor et al. [20] conducted a large-sample study, revealing that Cronbach's *α* coefficient for the SUS was 0.91. This scale uses a Likert 5-point scale and a score of 0–100. According to previous research results [21], SUS scores ranging from 50.9 to 71.4 are considered acceptable, those from 71.4 to 85.5 are considered good, and those from 85.5 to 90.9 are considered excellent.

### 2.5. Statistical Analysis

The collected data were analyzed using SPSS 26.0. The general characteristics and observed variables were analyzed using descriptive statistics, and the Kolmogorov–Smirnov test was used to test for normality. The Levene test was used for variance homogeneity, and the *t*-test or nonparametric tests were used for comparisons. The significance level was set to 0.05. The open questions were analyzed using NVivo 12.0 by classifying and thematizing the responses and counting the response frequencies [22].

### 2.6. Study Ethics

This study passed an ethical review by the Medical Ethics Committee of Southern Hospital, Southern Medical University. All survey responses were anonymous. There were no student names or identification (ID) on the questionnaires, and the teachers were not present when the students were filling out the questionnaires. Any patient information in the system was desensitized and anonymized.

## 3. Results

### 3.1. Nursing Information System Questionnaire Scores

**Figure 2:**
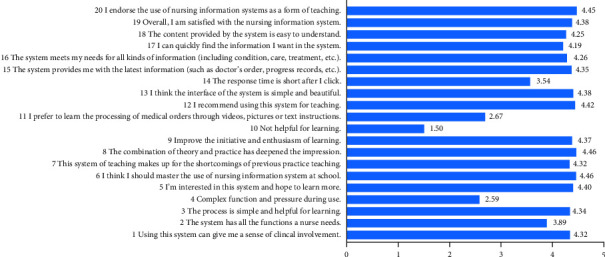
Strongly disagree = 1, disagree = 2, neutra1 = 3, agree = 4, strongly agree = 5.

Overall, 542 questionnaires were recovered, and 64 invalid questionnaires were excluded, including 45 with missing data, 17 with repeated options, and two with inconsistent data, representing an effective response rate of 88.19% (Figure 2).

### 3.2. SUS Score

In total, 544 scales were recovered, with 14 invalid scales excluded, including 10 with missing data, two with repeated data-filling, and two with identical answers. The effective response rate for the scale was 97.43%. After the calculations, the mean score was 72.625 ± 13.091 (mean ± standard deviation), the median was 72.500, and the range was 27.5–100.0. Statistically significant differences were found regarding the total scores on the SUS for the classes in different years, with the third-year students scoring higher than the second-year students (Table 1).

### 3.3. Open Questions

A total of 369 benefits were sorted and coded into 465 nodes, while 241 suggestions were obtained and coded into 276 nodes (Table 2).

## 4. Discussion

### 4.1. The Nursing Simulation Teaching Information System Can Meet the Needs of Nursing Students for Clinical Information, Help Students to Become Familiar with the Clinical Nursing Workflow, and Integrate Theory with Practice

In this study, 95.6% of the students believed that the system would provide them with the latest health-related information, such as medical advice and disease course records. Additionally, 91% of the students believed that the system provided information regarding patients' conditions, treatment methods, and nursing measures.

In the open question on the benefits of the nursing simulation teaching information system, some students expressed that they had the opportunity to use the system by themselves, while others stated that it helped them to understand the clinical nursing workflow in advance. Some of the responses are as follows:

Student 4: “Knowing how to use the nurse station and information system will help me master it in clinical practice in the future and enhance my confidence.”

Student 89: “I have an intuitive feeling about the operational advancement of clinical nursing. Using an electronic version can save more time and is more convenient. I can feel the convenience of information.”

Student 200: “The zero-distance contact with the nursing simulation teaching information system is very novel and deepens the impression of learning.”

This is the first time that students have had direct access to complete patient information in school. Students can browse and search relevant patients' information in the simulation system at will, process medical orders, fill in nursing assessment forms, and other perform operations, which helps students quickly become familiar with the clinical nursing workflow. In hospitals, students do not have an ID for the HIS, so they cannot log in and operate the system. The simulation system can be practiced repeatedly, which helps students quickly adapt to clinical practice.

### 4.2. Nursing Students Generally Support the Use of Nursing Simulation Teaching Information Systems in Teaching, Which can Improve Students' Learning Initiative and Interest and Deepen Their Learning Impression

A total of 98.5% of students approved of the use of the nursing simulation teaching information system, and 92.5% agreed that the nursing information system helped make up for the shortcomings of the previous teaching method.

The application of HISs has been popularized in hospitals. The corresponding teaching is very important, but it is missing [23, 24]. The results of this study demonstrated that when the nursing simulation teaching information system combined theory with practice, it improved the learning experiences of students (98.1%), created a sense of clinical reality (93.7%), and improved students' motivation and enthusiasm (94.3%). Generally, information technology can increase students' interest and motivation by simulating clinical work situations [25]. This is one of the reasons why the students in this study praised and recommended the system.

Carrying out nursing information technology education in colleges and universities has become popular [26]. HISs not only reduce cost and time but also reduce medical errors and patient mortality, promote nursing workflow, and improve the quality of nursing services for patients [27, 28]. Although the clinical application of HISs still faces many obstacles, including imperfect systems, data loss, information security problems, and a lack of systematic training [29, 30], such systems play an important role in clinical practice. In a previous study [31], Yu et al. selected six nursing colleges to conduct a questionnaire survey, and the results showed that the nursing information skills of nursing master's students were below the medium level, indicating nursing colleges should pay more attention to the teaching and adoption of HISs before nursing student internships. In this study, 97.5% of nursing students believed that they should master HISs use while in school.

### 4.3. Comparison with Existing Software

To the best of our knowledge, there is no report on similar HISs that can be used for laboratory simulation teaching, and there are no similar software products in the market. When building our nursing simulation teaching center, our original intention was to purchase the existing software. However, after contacting several companies, we were unsuccessful. In addition, we tried out software from other companies', such as Laerdal's vSim and DxR Nursing Select. The advantage of this software is that it allows students to focus on clinical thinking training [32, 33], but it is limited to the virtual simulation of a single case of a disease rather than the overall information system. This software lacks dynamic changes in cases from admission to discharge. In clinical practice, patients' situations are constantly changing, so the contents of the nursing assessment and nursing problems often differ. Therefore, the dynamic changes of cases should be integrated into the simulation teaching to help students improve their clinical ability and critical thinking skills.

However, the functions of the existing HIS in the hospital are too large and complex and involve different departmental modules of the hospital. In addition, for ethical reasons, it is difficult to directly use the hospital's HIS and its data during simulation teaching.

Nowadays, the hardware facilities of simulation teaching centers (simulation hospitals) built by universities or hospitals are becoming increasingly perfect and high-end. Computer-human patient simulators and digital virtual simulation facilities are routinely equipped. However, they lack a simulation teaching management system like the hospital HISs. It is a “central system” that improves how simulation centers are run. Without this central management system, each simulation unit can only conduct repeated training and assessments around a single case. It is difficult to achieve real “simulation” and create actual clinical scenarios.

### 4.4. Comparison with Existing Teaching Models

Nurses need to skillfully use HISs in their daily nursing work, which can actively help nurses to provide effective, safe, and high-quality nursing services for patients [34]. Studies have shown that training HISs users can help them make better use of such systems [35]. However, because there was no simulation HISs for teaching before, nursing students did not learn how to operate HISs and nurse stations during the school day. In China, HISs are introduced in the knowledge expansion section of the teaching material, which is not included in the formal teaching content. At the hospital internship stage, because the interns are not regular employees, the hospital will not assign students IDs for the HISs. Students can only see the teacher's demonstration and cannot operate the system by themselves.

After the completion of the system development, we added the learning content and practice of HISs to the fundamentals of nursing courses from 2020. Each nursing student has a system ID and can log in at any time to understand the information about the patients assigned by the teacher. Students can also process medical orders, fill in nursing assessment forms, write nursing records, and complete other operations online. At present, this system has been used to assist in the teaching of multiple courses, including the fundamentals of nursing, health assessment, implementation of nursing plans for senior nursing students, and emergency and critical care. It is expected to become more popular in the future and become a necessary software support system for nursing teaching.

### 4.5. Further Optimization of the Nursing Simulation Teaching Information System

Most nursing students were satisfied with the content, interface, reactivity, and learnability of the system, and 97.2% were satisfied with the system overall. The mean score on the SUS was 72.625 ± 13.0907, demonstrating that the evaluations of the system were good but that the system requires further optimization. The system developed by our team is still in the exploration stage, with 55.6% of the students mentioning that it was sometimes unresponsive. This was one of the factors hindering nursing staff from using HISs, as pointed out by Handayani et al.[34]. In the open questions, some students also suggested adding a “help” button and an annex for drug descriptions. In terms of teaching, it was suggested that the teacher should demonstrate the operation in advance, after which the students would be divided into groups to simulate and operate it by themselves, assign tasks after class to consolidate the knowledge learned in class, and increase the time for students to operate the system by themselves. Some students suggested supplementing the system training with teaching videos or instructional books to improve learning efficiency. Meanwhile, two students suggested that the curriculum arrangement for the system teaching should be moved to the end of the semester because the system practice should be based on certain theoretical knowledge to make it more similar to clinical practice. The results of this study revealed that the junior nursing students gave higher SUS scores than the sophomores because, compared with the sophomores, the juniors had richer professional knowledge and clinical practice experience and a more intuitive understanding and evaluation of the nursing simulation teaching information system's application. Booth et al. [37] suggested that the design and application of electronic information systems should consider the composition of team members, which should be suitable for those students who are still in the formative stage of their education or professional knowledge. All these factors provide direction and suggestions for optimizing the system in the future.

### 4.6. Limitations of the Study

A self-compiled questionnaire was used as a survey in this study. Although expert consultations and demonstrations were carried out during the early stages, there may still have been deviations in the results. Open questions were used as a supplement to the survey content to reduce any bias. None of the student participants in this study had participated in a clinical internship, and the sample was relatively concentrated. Nursing education can be carried out in the future based on the nursing simulation teaching information system improvements made after this study, and collegiate partnerships will be considered to expand the audience.

## 5. Conclusion

This study designed and extended a nursing simulation teaching information system and improved the corresponding teaching models, leading to a high degree of satisfaction among nursing students. The nursing students proposed suggestions about the teaching format and system optimization, which provides a reference for future education using the nursing simulation teaching information system. Based on this, the simulation teaching information system can be popularized and applied in the teaching of other medical majors, such as clinical medicine, clinical pharmacy, and medical testing technology, to make medical education more similar to clinical practice.

## Figures and Tables

**Table 1:** Scale scores of the different year classes (*N* = 530).

Grade	*N*	Mean ± SD	Kolmogorov–smirnov test	Levene test	Wilcoxon rank-sumtest
Third-year	220	73.898 ± 13.7412	0.018	0.070	0.041
Second-year	310	71.806 ± 12.5590	0.004		

**Table 2:** Nurse students' benefits and suggestions for the system's teaching.

	Theme	Statement (%)
Gains	(i) Access to skills	44.52
	Familiar with the operation process of the system	
	Learn about the daily work of nurses	
	Acquire clinical nursing knowledge	
	(ii) Clinical significance	37.42
	The system is convenient and close to clinical	
	The intuitive experience of clinical operation and a sense of clinical substitution	
	(iii) Realize the importance of information technology	8.39
	(iv) Deepen the impression of the learning content, and the teaching effect is good	8.17
	(v) No clear gain experience is given	1.51

Suggestions	(i) To optimize the system	52.17
	Resolve system lag	
	Add patients' cases	
	Improve the interface	
	(ii) Complete the system instructions for learning videos, PPT, manuals, and so on	11.59
	(iii) Give students more time to operate independently offline	7.61
	(iv) Course advice	7.49
	Guide students to learn relevant knowledge before class	
	Slow down and focus on the main points when teaching	
	Adopt group learning	
	Assign tasks after class	
	Adjust the course to the later stage	
	(v) No specific recommendations were made	4.12

## Data Availability

The data used to support the findings of this study are available from the corresponding author upon reasonable request.
